# Retropubic versus transobturator slings: Medium‐term satisfaction and overactive bladder outcomes

**DOI:** 10.1002/ijgo.70849

**Published:** 2026-02-04

**Authors:** Erika Gandelsman, Jonatan Neuman, Réka Fábián‐Kovács, Talia Friedman, Menahem Neuman, Benjamin Feiner

**Affiliations:** ^1^ Department of Obstetrics and Gynecology Hillel Yaffe Medical Center Hadera Israel; ^2^ Rappaport Faculty of Medicine Technion Israel Institute of Technology Haifa Israel; ^3^ Faculty of Medicine Semmelweis University Budapest Hungary; ^4^ Department of Obstetrics and Gynecology; Sheba Medical Center Tel Aviv University Tel Aviv Israel; ^5^ Faculty of Medicine Ariel University Ariel Israel; ^6^ Urogynecology and Pelvic Floor Medicine, Medical School Bar‐Ilan and the Negev Universities Haifa Israel; ^7^ Assuta Medical Centers Tel Aviv Israel

**Keywords:** mid‐urethral sling, overactive bladder, patient reported outcomes, patient satisfaction, stress urinary incontinence, tension‐free vaginal tape, transobturator tape

## Abstract

**Objective:**

This study compares medium‐term outcomes of retropubic tension‐free vaginal tape (TVT) and transobturator tape (TOT) for stress urinary incontinence (SUI), focusing on patient satisfaction and overactive bladder (OAB) symptoms.

**Methods:**

This prospective, single‐surgeon cohort study included women with SUI who underwent TVT or TOT at a single center (July 2021–October 2022). Follow‐up was conducted at 26–41 months through chart review and patient interviews. Satisfaction was rated on a 0–100% global scale (≥75% = satisfied). Outcomes and complications followed International Continence Society criteria. Continuous variables were compared with the Mann–Whitney *U*‐test and categorical variables with Fisher's exact test. Sensitivity analysis addressed the effects of loss to follow‐up. Statistical significance was set at *P* < 0.05.

**Results:**

Fifty‐three women (25 TVT, 28 TOT) completed follow‐up. Satisfaction (≥75%) was reported by 88.0% of TVT and 89.3% of TOT patients (*P* ≈ 1.00). Sensitivity analyses assuming all lost patients were satisfied or unsatisfied did not alter statistical significance (*P* ≈ 1.00 and *P* = 0.54, respectively). Among women with pre‐existing overactive bladder, improvement in symptoms occurred in 78.3% (18/23) of TVT and 61.1% (11/18) of TOT patients (*P* = 0.47). One bladder perforation occurred in the TVT group; other complications were infrequent and similar between groups.

**Conclusions:**

Both TVT and TOT provided high satisfaction and improvement in OAB symptoms at 26–41 months, with low complication rates. These findings suggest that both procedures remain safe and effective in the medium term, reinforcing their established role in the surgical management of SUI.

## 1 INTRODUCTION

Stress urinary incontinence (SUI) is the most common form of female urinary incontinence, affecting up to 40% of women and impairing quality of life.^1^ Since the introduction of retropubic tension‐free vaginal tape (TVT) in 1996,^2^ mid‐urethral slings (MUS) have become the gold standard for surgical management of SUI. The transobturator tape (TOT), described later, was developed to reduce complications associated with the retropubic route, particularly bladder perforation and serious vascular injury.^3^, ^4^

High‐quality randomized controlled trials and meta‐analyses have established that both approaches are highly effective, with cure rates exceeding 80%.^1^, ^4^, ^5^ Complication profiles differ slightly, with TVT more often linked to bladder perforation and voiding dysfunction, while TOT is associated with transient groin or thigh pain.^1^, ^6^, ^7^ Long‐term outcomes are generally favorable for both,^1^ although evidence beyond short‐term follow‐up (≤12 months) is limited. However, recent systematic reviews emphasize that despite the widespread use of synthetic mid‐urethral slings, high‐quality long‐term data remain limited, and reported outcomes beyond five years are heterogeneous, particularly regarding late complications. This underscores the need for contemporary studies reporting patient‐centered medium‐term outcomes to further contextualize the safety and effectiveness of these procedures.^8^

Most randomized trials have focused on objective cure rates, whereas patient‐centered outcomes such as satisfaction and overactive bladder (OAB) symptom changes ‐ both improvement in pre‐existing urgency and the risk of de novo urgency ‐ are less consistently reported, partly because many studies have excluded women with pre‐existing OAB.^1^ These outcomes are clinically important, particularly for women with mixed incontinence.

This study presents prospective, single‐surgeon data with 26–41 months of follow‐up, comparing TVT and TOT. All procedures were performed using the Serasis partially absorbable sling, designed to reduce permanent foreign body load; its use provides additional context to the comparison of retropubic and transobturator approaches. By focusing on patient satisfaction and OAB symptom changes, our work complements existing randomized evidence and addresses gaps in medium‐term, patient‐centered outcomes. We hypothesized that both approaches would yield comparable satisfaction and safety profiles.

## 2 METHODS

This was a quasi‐randomized prospective cohort study conducted at Assuta Medical Centers (Israel) between July 2021 and October 2022. Women with SUI who underwent either TVT or TOT were eligible. Women with incomplete medical records or who declined to participate were excluded. The surgical approach was assigned by simple random allocation (coin flip) performed immediately prior to surgery, independent of patient characteristics.

All procedures were performed by a single experienced surgeon under general anesthesia, using the Serasis mid‐urethral sling (Serag‐Wiessner, Naila, Germany), a soft, partially absorbable tape composed of polypropylene and polyglycolic acid‐caprolactone. TVT was performed via the retropubic route as originally described by Ulmsten et al.^2^ TOT was performed using De Leval's inside‐out technique, with a medialized needle trajectory subsequently modified to reduce risk of thigh pain.^4^, ^9^

Definitions followed the International Continence Society (ICS) recommendations.^10^ SUI was defined as patient‐reported involuntary urine leakage during physical activity, coughing, or sneezing. OAB was defined as patient‐reported urinary urgency, with or without urgency incontinence, increased daytime frequency, and/or nocturia in the absence of urinary tract infection. OAB severity was graded as mild, moderate, or severe, based on patient‐reported symptom burden. Postoperative bladder outlet obstruction (BOO) was defined as patient‐reported weak urinary stream, hesitancy, intermittency, or incomplete bladder emptying after the surgery. Pelvic organ prolapse (POP) was defined as patient‐reported vaginal bulging, pressure, or protrusion from the vaginal opening. Urinary tract infection (UTI) was defined by a positive urine culture.

Follow‐up was conducted 26–41 months postoperatively through chart review and patient interviews. Patient satisfaction was assessed by a self‐reported global rating (0%–100%), reflecting overall outcome, expectations, recovery, and adverse effects. Scores ≥75% were classified as satisfied and < 75% as unsatisfied. A threshold of ≥75% was chosen to represent high, clinically meaningful satisfaction and to distinguish substantial improvement from partial benefit. Although this global scale is not a validated patient‐reported outcome measure, its format is conceptually aligned with validated single‐item global impression instruments such as the PGI‐I, which has demonstrated strong construct validity in pelvic floor surgery.^11^ Patients were also asked about unwanted short‐ and long‐term side effects, including routine questioning regarding thigh and groin pain.

Continuous variables were assessed for normality using the Shapiro–Wilk test. Several variables, including age, parity, and duration of SUI, deviated from normality; therefore, all continuous variables were analyzed using the Mann–Whitney *U*‐test. Categorical variables were compared using Fisher's exact test. Sensitivity analyses addressed loss to follow‐up, assuming alternatively that all were satisfied or all were unsatisfied. Follow‐up duration was compared between groups using the Mann–Whitney *U*‐test. Given the modest sample size and comparable baseline characteristics between groups, no multivariable adjustment for potential confounders (e.g., baseline OAB severity) was performed. Statistical significance was set at *P* < 0.05. Analyses were performed using jamovi (version 2.6).^12^

The study was approved by the Assuta Medical Center Research Ethics Committee (0101‐20‐ASMC). Written informed consent was obtained from all participants prior to inclusion.

## 3 RESULTS

Sixty women were included: 30 underwent TVT and 30 underwent TOT. Follow‐up ranged from 26 to 41 months postoperatively. Seven patients (five TVT, two TOT) were lost to the satisfaction assessment and were unavailable for the medium‐term follow‐up; therefore, they did not contribute OAB outcome data. The median follow‐up duration was 3.04 years (interquartile range 0.17) in the TVT group and 3.02 years (interquartile range 0.13) in the TOT group, with no significant difference between groups (*P* = 0.72). Sensitivity analyses were performed only for the satisfaction outcome.

Baseline characteristics including age (*P* = 0.38), parity (*P* = 0.38), duration of SUI (*P* = 0.32), and comorbidities were comparable between groups (all *P* > 0.05; Table 1).

**TABLE 1:** Baseline characteristics of TVT and TOT groups.

Characteristics	TVT *n* = 30	TOT *n* = 30	*P*‐value
Age, years	53	57.5	0.38^a^
Parity, *n*	3	3	0.38^a^
SUI duration, years	2.0	1.5	0.32^a^
Comorbidities, *n*, %			
OAB	22, 73.3%	17, 56.7%	0.28^b^
UTI	1, 3.3%	4, 13.3%	0.35^b^
HTN	11, 36.7%	5, 16.7%	0.14^b^
DM	5, 16.7%	3, 10.0%	0.71^b^
Previous surgeries	5, 16.7%	2, 6.7%	0.42^b^

*Note*: Values are presented in median or *n* (%). Statistical analysis.

Abbreviations: DM, diabetes mellitus; HTN, hypertension; OAB, overactive bladder; SUI, stress urinary incontinence; TOT, transobturator tape; TVT, retropubic tension‐free vaginal tape; UTI, urinary tract infection.

^a^ Mann–Whitney *U*‐test.

^b^ Fisher exact test.

**FIGURE 1:**
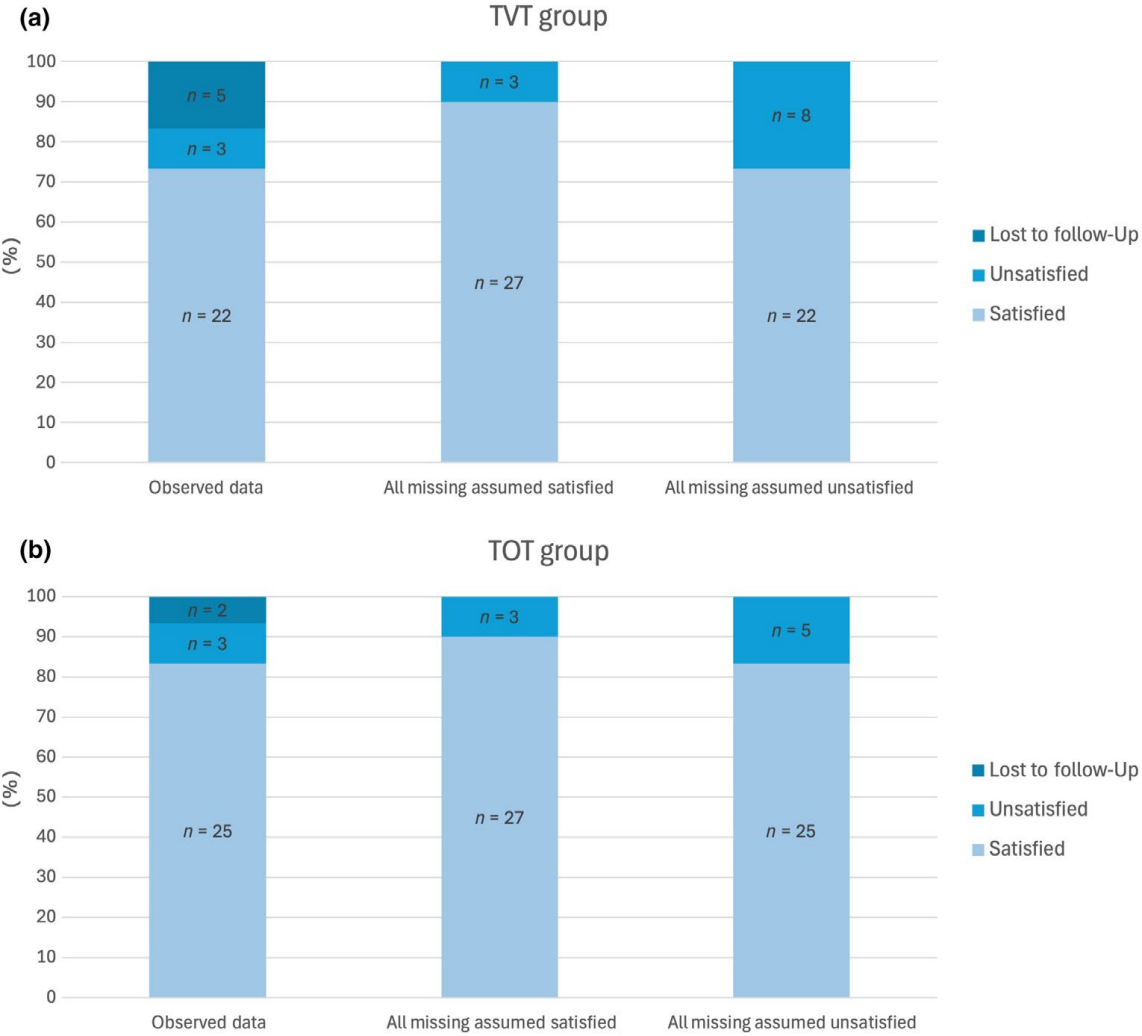
Satisfaction rates with sensitivity analyses for missing data. (a) Retropubic tension‐free vaginal tape (TVT) group. (b) Transobturator tape (TOT) group. Patient satisfaction was rated on a self‐reported global scale (0–100%) reflecting overall outcome and recovery experience; scores ≥75% were classified as “satisfied,” and <75% as “unsatisfied.”

Satisfaction (≥75%) was reported by 22 of 25 (88.0%) in the TVT group and 25 of 28 (89.3%) in the TOT group (*P* ≈ 1.00; odds ratio 0.88, 95% confidence interval 0.15–4.58; Table S1). Sensitivity analyses assuming all missing patients were satisfied or unsatisfied did not alter statistical significance (*P* ≈ 1.00 and *P* ≈ 0.54, respectively; Figure 1).

**FIGURE 2:**
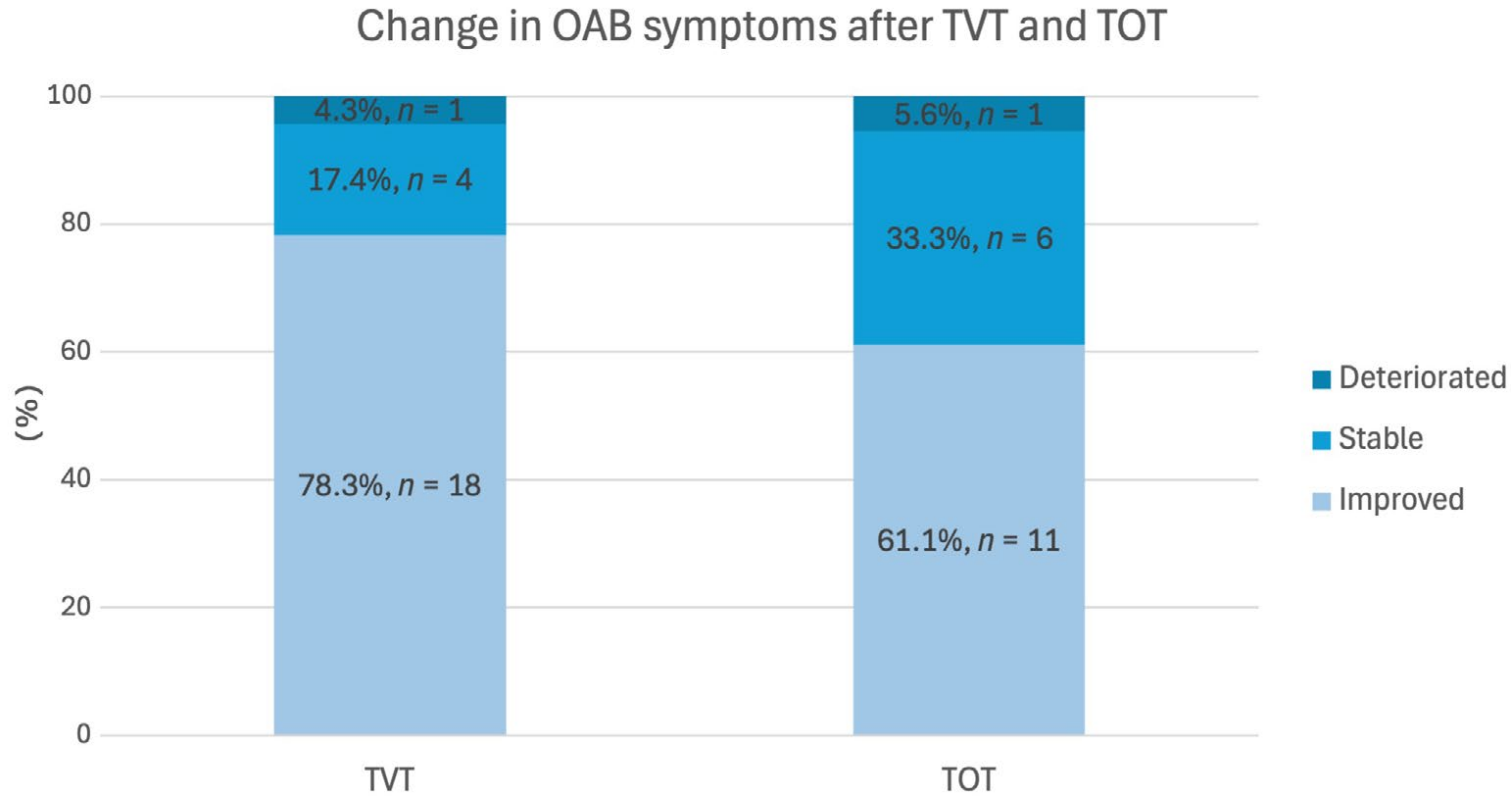
Change in overactive bladder (OAB) symptoms after retropubic tension‐free vaginal tape (TVT) and transobturator (TOT) slings. Improvement, stability, and worsening were self‐reported by patients with pre‐existing OAB at medium‐term follow‐up (26–41 months).

Among women with pre‐existing OAB, improvement after surgery was reported in 18 of 23 (78.3%) TVT patients and 11 of 18 (61.1%) TOT patients, while symptoms remained stable in four (17.4%) and six (33.3%) patients and worsened in one (4.3%) and one (5.6%) patient, respectively (*P* = 0.47; Figure 2; Table S2). One patient in the TVT group developed de novo OAB.

Intraoperative and postoperative complications were infrequent (Table 2). One bladder perforation occurred during TVT, managed by immediate needle re‐insertion and 4 days of urethral catheterization, with full recovery. No perforations occurred with TOT. Early postoperative BOO occurred in three TVT and two TOT patients; tape release was required in one and two cases, respectively. Temporary catheterization was needed in two TVT patients and none in the TOT group. One woman in the TOT group reported transient thigh pain, which resolved without sequelae. No cases of groin pain were observed in either group. Mid‐term UTI occurred in one patient in each group. At long‐term follow‐up, there were no significant differences between groups in rates of recurrent SUI, OAB, POP, UTI, or BOO (all *P* > 0.05).

**TABLE 2:** Complications after TVT and TOT.

Complications	TVT	TOT	*P*‐value
Intraoperative			
Bladder penetration	1, 3.3%	0, 0.0%	1.00^a^
Major vascular injury	0, 0.0%	0, 0.0%	—
Early postoperative			
BOO	3, 10.0%	2, 6.7%	1.00^a^
Tape release	1, 3.3%	2, 6.7%	1.00^a^
Temporary catheterization	2, 6.7%	0, 0.0%	0.49^a^
UTI	0, 0.0	0, 0.0%	—
Mid post‐operative			
UTI	1, 3.3%	1, 3.3%	—
Thigh pain	0, 0.0%	1, 3.3%	1.00^a^
Long‐term (26–41 months)			
SUI	3, 12.0%	5, 17.9%	0.70^a^
OAB	6, 24.0%	9, 32.1%	0.51^a^
POP	4, 16.0%	4, 14.3%	1.00^a^
UTI	1, 4.0%	1, 3.6%	1.00^a^
BOO	1, 4.0%	0, 0.0%	0.46^a^
Further surgery			
Sling removal	0, 0.0%	2, 6.7%	0.49^a^
TVT	—	1, 3.3%	1.00^a^

*Note*: Values are *n* (%). Statistical analysis.

Abbreviations: BOO, bladder outlet obstruction; OAB, overactive bladder; POP, pelvic organ prolapse; SUI, stress urinary incontinence; TOT, transobturator tape; TVT, retropubic tension‐free vaginal tape; UTI, urinary tract infection.

^a^ Fisher exact test.

## 4 DISCUSSION

In this prospective single‐surgeon cohort, both retropubic and transobturator MUS demonstrated high and comparable satisfaction rates at 26–41 months of follow‐up, with low complication rates. These findings are consistent with the TOMUS randomized trial, which reported similar subjective and objective success at 12 months,^5^ and with the Cochrane review, which concluded that both routes are effective and safe, with only nuanced differences in complication patterns.^1^ Our results extend this equivalence into the medium term, suggesting that most women maintain high satisfaction for up to 3 years after surgery. These outcomes were achieved using a partially absorbable sling; although our study was not designed to compare sling materials, the findings describe the medium‐term performance of the Serasis sling within this cohort.

Recent long‐term evidence further contextualizes these findings. Large population‐based analyses have shown that reoperation rates after mid‐urethral sling surgery increase gradually over time, reaching approximately 5% after more than 10 years, with patient‐related factors such as a pre‐existing overactive bladder identified as important predictors of reoperation rather than the surgical route itself. In parallel, extended follow‐up of randomized cohorts has demonstrated durable patient‐reported improvement up to 15–16 years after surgery, with no significant differences between retropubic and transobturator approaches.^13^, ^14^

The sensitivity analyses addressing attrition bias strengthen confidence in these findings. Even under the conservative assumption that all patients lost to follow‐up were dissatisfied, overall satisfaction remained within the 60–80% range reported in long‐term series.^15^, ^16^ Few studies have accounted for attrition in this way, and this strengthens the novelty of our approach.

Our study also provides data on OAB symptom change. Among women with pre‐existing OAB, improvement was observed in 78.3% of the TVT group and 61.1% of the TOT group, with no significant difference between the two approaches. To account for baseline variability, OAB outcomes were analyzed as within‐patient changes from each woman's own preoperative status rather than as absolute severity levels. The study was not powered for severity‐stratified subgroup analyses.

Because many prior trials excluded cases with detrusor overactivity or OAB, direct comparisons of urgency outcomes across studies are limited. Reported rates of de novo urgency after MUS are typically 8–12% with no significant difference between surgical routes,^1^ whereas de novo OAB occurs at lower rates (approximately 6–8%).^17^ In our cohort, de novo OAB was infrequent (1.7%), likely reflecting our clinically focused definition, which captured only clearly new and bothersome symptoms, the interview‐based follow‐up, and the modest sample size. Some early postoperative urgency symptoms might also be transient and resolve before medium‐term assessment, which could contribute to the low observed rate.

Our observations also align with data showing that urgency often persists despite successful treatment of the stress component,^18^ underscoring that OAB patterns after MUS are more variable and less predictable than stress continence outcomes. These findings support the observation that the risk of clinically meaningful new urgency after MUS is generally low.

We reviewed baseline characteristics in relation to OAB improvement, but no consistent patterns emerged. As the study was not powered for stratified analyses and the preoperative OAB severity scale was pragmatic rather than validated, further subgroup analysis was not pursued. Follow‐up duration was comparable between groups, making it unlikely that differences in the timing of the medium‐term assessment contributed to the observed OAB outcomes.

In examining the relationship between medium‐term symptoms and satisfaction, we found that 4/8 women with recurrent SUI, 13/15 women with persistent OAB, and 3/6 women with POP still reported satisfaction (≥75%). These findings suggest that individual medium‐term symptoms did not necessarily preclude high satisfaction, likely reflecting the multifactorial nature of postoperative outcome perception.

Complication rates were low and procedure specific. One bladder perforation occurred in the retropubic group, in line with published estimates of 3–5%.^16^ One case of transient thigh pain was reported in the TOT group, consistent with the known complication profile of the transobturator route and resolved without sequelae.^15^ No serious late complications were observed. All were minor and resolved without sequelae, underscoring the safety of both procedures when performed by an experienced surgeon.

Strengths of this study include its prospective design, standardized surgical technique by a single surgeon, and medium‐term follow‐up with a focus on patient‐centered outcomes. Limitations include the modest sample size, lack of validated quality‐of‐life questionnaires, and single‐center design. The study was not powered for severity‐stratified analyses; however, baseline characteristics including pre‐existing OAB were similar between groups, reducing the likelihood of confounding. Several additional considerations merit mention. Urodynamic studies were not routinely performed, limiting objective confirmation of SUI and the ability to identify detrusor overactivity. OAB symptoms were assessed pragmatically rather than with validated, standardized tools. Although all women were evaluated within the medium‐term window of 26–41 months, stratified analyses by follow‐up interval were not feasible.

## 5 CONCLUSION

Both TVT‐retropubic and TOT provided high satisfaction and durable continence outcomes at 26–41 months, with no significant differences between groups. OAB symptoms improved in many women with pre‐existing urgency, and only one de novo case was observed. Complication rates were low and manageable, and all procedures were performed with a partially absorbable sling, supporting its safety in routine practice. These results reinforce international evidence that both techniques remain safe and effective surgical treatments for SUI, with procedure choice guided by patient characteristics and surgeon expertise.

## AUTHOR CONTRIBUTIONS

EG drafted the manuscript. JN coordinated data collection. RFK performed statistical analyses and contributed to data interpretation. TF contributed to manuscript revision. MN contributed to study conception, discussion and literature review. BF contributed to clinical oversight and critical revision of the manuscript. All authors reviewed and approved the final version and agree to be accountable for all aspects of the work.

## FUNDING INFORMATION

This research received no specific grant from any funding agency in the public, commercial, or not‐for‐profit sectors.

## CONFLICT OF INTEREST STATEMENT

The authors have no conflicts of interests to declare.

## Supporting information


Data S1.

## Data Availability

Research data are not shared.
