# Supplementary material for: Retropubic versus transobturator slings: Medium‐term satisfaction and overactive bladder outcomes
Source: Int J Gynaecol Obstet. 2026 Feb 4;174(1):391–6. doi: 10.1002/ijgo.70849 (PMC13278633; doi:10.1002/ijgo.70849)
Supplement: Supplementary file 1 — Data S1. [file IJGO-174-391-s001.docx]

### Supplementary Material - Additional Tables

The following supplementary tables present detailed results of sensitivity analyses for satisfaction rates and changes in overactive bladder symptoms, provided to support the findings presented in the main text.

**S1. Sensitivity analyses of satisfaction rates**

| Satisfaction level, n (%) | TVT  n=30 | TOT  n=30 | p-value |
| --- | --- | --- | --- |
| Satisfied | 22 (73.3%) | 25 (83.3%) | 1.00^a^ |
| Unsatisfied | 3 (10%) | 3 (10%) |  |
| Lost to follow-up | 5 (16.6%) | 2 (6.6%) |  |
| Assuming all missing satisfied  Satisfied  Unsatisfied | 27 (90%)  3 (10%) | 27 (90%)  3 (10%) | 1.00^a^ |
| Assuming all missing unsatisfied  Satisfied  Unsatisfied | 22 (73.3%)  8 (26.6%) | 25 (83.3%)  5 (16.6%) | 0.54^a^ |

S1 presents satisfaction rates under three alternative assumptions regarding patients lost to follow-up.Statistical analysis: ^a^Fisher’s exact test. TOT, transobturator tape; TVT, retropubic tension-free vaginal tape.

**S2. Change in OAB symptoms after TVT and TOT**

| Irritative symptom change,  n (%) | TVT  n=23 | TOT  n=18 | p-value |
| --- | --- | --- | --- |
| Improved | 18 (78.3%) | 11 (61.1%) | 0.47^a^ |
| Stable | 4 (17.4%) | 6 (33.3%) |  |
| Deteriorated | 1 (4.3%) | 1 (5.6%) |  |

S2 summarizes postoperative changes in OAB symptoms among patients who reported pre-existing OAB before surgery. Statistical analysis: ^a^Fisher’s exact test. OAB, Overactive bladder; TOT, transobturator tape; TVT, retropubic tension-free vaginal tape.
